# Perceived Changes in Family Life During COVID‐19: The Role of Family Size

**DOI:** 10.1111/fare.12579

**Published:** 2021-09-15

**Authors:** Elena Canzi, Francesca V. Danioni, Miriam Parise, Giulia Lopez, Laura Ferrari, Sonia Ranieri, Raffaella Iafrate, Margherita Lanz, Camillo Regalia, Rosa Rosnati

**Affiliations:** ^1^ Università Cattolica del Sacro Cuore Milan Italy

**Keywords:** COVID‐19 pandemic, family life, family size, negative and positive changes

## Abstract

**Objective:**

The current study was aimed at exploring Italian parents' perceived negative and positive changes in family life during the COVID‐19 pandemic, taking into account the role of the stage of the family life and family size.

**Background:**

During the emergency of the COVID‐19 pandemic, millions of families drastically changed their daily life and routines. Little evidence exists on how family characteristics, such as family size or presence of children, are related to families' experience of family change.

**Method:**

A large sample of 1,407 Italian parents (70.1% mothers) filled in an anonymous online survey during the third week of the lockdown period (between March 30 and April 7, 2020).

**Results:**

Results showed that parents reported perceiving more positive changes than negative ones, especially in terms of feeling more emotionally close to their children and spending more fun time with them. Interestingly, parents with two or more children reported more positive changes in family life compared with parents who had one child, showing a greater relational regenerative capacity in the face of COVID‐19 lockdown.

**Conclusion and Implications:**

Investing in family relationships, especially for larger families, is an effective coping strategy to deal with traumatic situations and promote positive family changes in stressful situations.

During the emergency of COVID‐19 pandemic, millions of families were forced to drastically change their daily life and routines. Italy is one of the countries severely affected by this health emergency. As of August 25, 2021, there were 4,502,396 confirmed cases and 128,914 deaths.

For a period of time lasting almost 3 months, Italian families practiced physical distancing by isolating themselves at home and restricting movements and interactions. Indeed, the Italian government, starting on March 11, 2020, imposed progressive restriction measures, forcing Italian families into lockdown to control the spread of the virus. Group activities, social gatherings, and outdoor activities were strongly limited or prohibited. Any kind of unessential business was forced to close and, when possible, working remotely from home was strongly encouraged. Moreover, schools and educational services were closed.

These drastic measures were crucial to contain the pandemic and protect the health system from being overwhelmed. However, the measures profoundly affected people's quality of life (Brooks et al., 2020). For parents in particular, this resulted in a increase in time spent with children at home and with more direct provision of their children's educational (i.e., about 8.5 million children homeschooled in Italy during the pandemic; Fontanesi et al., 2020) and childcare needs, without having access to formal and informal care support.

The COVID‐19 pandemic is a “perfect storm of stressors” (Walsh, 2020, p. 7) for families who experience a pervasive sense of loss: the death of loved ones, loss of social and physical contact with significant others, loss of life plans and rituals, and loss of financial stability (Fraenkel & Cho, 2020; Walsh, 2020). There are expectations of long‐term, acute psychological reactions to these pandemic stressors, including posttraumatic stress disorders, emotional disturbances, and depressive disorders (Mucci et al., 2020). This situation poses a huge challenge to psychological resilience (Wang et al., 2020), which encourages researchers to explore how to help families coping with stressors linked to the pandemic.

Research so far has primarily focused on the individual consequences of the pandemic (e.g., Brooks et al., 2020; Casagrande et al., 2020), but little evidence exists on how families experienced daily life during this health emergency (Günther‐Bel et al., 2020). About one third of families have reported feeling very or extremely anxious about situations derived from the COVID‐19 lockdown (Statistics Canada, 2020). For a large number of parents, concerns related to the pandemic (e.g., financial worries, sadness for social isolation, concerns for children's well‐being) significantly affected their parenting (Lee & Ward, 2020). Indeed, parents reported more conflicts with their children and increased discipline (Lee & Ward, 2020).

In a study carried out by Fontanesi and colleagues (2020) with 1,126 Italian parents, most participants reported relevant negative changes in their children's well‐being and an increase in their authoritarian parenting style. Another study carried out in Italy by Spinelli and colleagues (2020), which involved 854 parents with children aged 2 to 14 years, examined whether parental well‐being was linked to their children's well‐being: findings showed that the higher the difficulty for parents to face the restrictive measures, the greater were parental stress and the children's behavioral and emotional problems. Interestingly, living in at‐risk zones or being in contact with the virus effects did not significantly influence family well‐being.

On the positive side, studies also found that parents reported spending more time in activities with their children, as well as experiencing higher feelings of closeness and warmth compared with the pre‐lockdown period (Lee & Ward, 2020). Similarly, taking care of children was found to be associated to increased positive feelings and decreased negative feelings (Lades et al., 2020). Moreover, a study by Günther‐Bel et al. (2020) interestingly found that parents reported more relational improvement than deterioration during the lockdown period, suggesting processes of resilience within families during the COVID‐19 pandemic as well.

The current exploratory study was aimed at evaluating parents' perceptions about how their family life changed, both negatively and positively, during the COVID‐19 lockdown phase. As already mentioned, Italy was severely affected by the pandemic, and Italian parents may represent a relevant target population when studying the impact of COVID‐19. Specifically, given that lockdown and stay‐at‐home orders are specific stressors of the COVID‐19 pandemic having a potential impact on family well‐being (Prime et al., 2020), we considered all families with cohabiting children. This is in line with recent studies considering the presence of cohabiting people as a possible risk or protective factor for mental health outcomes in the face of COVID‐19–related experiences (e.g., Fiorillo et al., 2020; Günther‐Bel et al., 2020).

Additionally, due to the high variability in how families may be affected by the COVID‐19 pandemic (Prime et al., 2020), we explored the role of some structural factors in determining how parents perceived any changes in their family's life. In particular, we considered the stage of the family life cycle and family size, which emerged as relevant in the psychosocial literature and also may play a role in how families face the pandemic. Indeed, families may differ in their ability to adjust to the stressors related to the pandemic depending on the stage of their life cycle. Previous research has documented that parenting stress generally decreases as children grow older (Neece et al., 2012; Williford et al., 2007). In the current study, we considered the following stages of the family life cycle: (a) parents with children under 13 years of age, (b) parents with adolescents aged between 13 and 17 years, and (c) parents of young adults aged between 18 and 34 years. This latter age range was adopted based on the fact that in the Italian context, young adults tend to cohabitate in the parental home for prolonged time (Carrà et al., 2014; Eurostat, 2020).

Moreover, family size is generally recognized as a risk factor for parents' well‐being, given the link between family size and higher levels of parenting stress (Gameiro et al., 2009; Hong & Liu, 2019; Östberg & Hagekull, 2000). However, a study on horse owners quarantined because of equine influenza in Australia in 2008 revealed that having one child, as opposed to no children, was associated with higher psychological distress, but having three or more children appeared to be a protective factor (Taylor et al., 2008). In line with this finding, a recent study by Fiorillo et al. (2020) has shown that living with a higher number of family members is a protective factor against the development of psychiatric symptoms during the lockdown imposed by COVID‐19 pandemic.

In exploring the association between these structural factors and perceived changes in family life, we controlled for parental gender. Indeed, according to existing evidence, the COVID‐related situation appears to differentially impact men and women, with women being more challenged in terms of psychological health (Liu et al., 2020), caregiving overload (Collins et al., 2020), and employment (Alon et al., 2020). Moreover, we also controlled for parent–child relationship satisfaction, based on the fact that relationship quality could be a resource or, on the contrary, a risk factor, in the process of appraisal and stress management (Prime et al., 2020). Finally, we included knowing people diagnosed with COVID‐19 as a covariate, given that recent studies have shown that this variable may put people at higher risk of experiencing distress (Barni et al., 2020; Rapelli et al., 2020).

In sum, the current study had two purposes: (a) to describe parents' perceptions about how their family life changed, both negatively and positively, during the COVID‐19 lockdown and (b) to explore whether and how family characteristics, such as the stage of the family life cycle and family size, were related to families' experience of family change controlling for parental gender, parent–child relationship satisfaction, and knowing people diagnosed with COVID‐19.

## method

### Participants

The study was part of a wider research project “The Family at the Time of COVID‐19,” which was carried out by the Family Studies and Research University Centre of the Università Cattolica del Sacro Cuore, which included a large sample of the Italian parents. In the current study, we considered information provided by parents who had at least one cohabitating child. The final sample of this study included 1,407 parents (men = 29.9%, *n* = 421; women = 70.1%, *n* = 986). In relation to age, 10.3% parents were younger than 34 years of age, 38% were between 35 and 44 years of age, 38.7% were between 45 and 54 years of age, and 13% were over 54 years of age. With regard to their place of residence, parents were distributed as follows: 45.3% of them in the north of Italy, 20.9% in the center, and 33.8% of participants lived in the south of Italy or on an island. With regard to the level of education, 10.3% had completed primary school, 56.5% had completed high school, and 33.2% had reached a university degree. Most parents were married (79.6%), 17% reported cohabiting with their partners, and 3.4% were separated or divorced. As for family size, 49.1% (*n* = 691) parents had one cohabiting child, 42.4% (*n* = 597) had two cohabiting children, and 8.5% (*n* = 119) had three or more cohabiting children. With regard to the stage of the family life cycle, we considered the age of the eldest child: 48.2% (*n* = 678) of parents cohabited with children aged below 13 years, 20% (*n* = 281) of parents cohabited with adolescents aged between 13 and 17 years, 31.8% (*n* = 448) of parents cohabited with young adults aged between 18 and 34 years.

Descriptive analyses showed that 27.6% (*n* = 388) of participants reported knowing at least one person who was sick because of COVID‐19 whereas 72.4% (*n* = 1,019) did not. Finally, at the time of data collection, 27% of parents worked at home, 16% of parents continued to work outside the home, and for 31.4% of parents' job activities were interrupted due to restrictive measures; 24.7% of parents were unemployed, and 0.9% were on maternity or medical leave.

### Procedure and Measures

The study was approved by the Ethics Committee of the Department of Psychology of Università Cattolica del Sacro Cuore (protocol number 15–20), and the study procedures followed the American Psychological Association ethical guidelines for human research (http://www.apa.org/ethics/code/). All respondents gave informed consent before participation in this study. Parents who enrolled in the study were asked to complete an anonymous online survey between March 30, 2020, and April 7, 2020, which was during the third week of the COVID‐19 pandemic response lockdown. The questionnaire included questions about demographic information and the following measures.

#### Perceived negative changes in family life

Participants were asked to respond to seven ad hoc items (from 1 = *strongly disagree*, to 5 = *strongly agree*), measuring different perceived negative changes in family life during the lockdown imposed by COVID‐19. Items examples are “We argue more” and “We have become more intolerant.” The items were used in two ways: for descriptive purposes, we considered each item separately, whereas for inferential purposes, we used a composite index computed by averaging the scores of the seven items. The scale showed good internal consistency (α = .86). Higher scores indicated higher perceived negative changes in family life.

#### Perceived positive changes in family life

Participants were asked to respond to six ad hoc items (from 1 = *strongly disagree*, to 5 = *strongly agree*), measuring different perceived positive changes in family life during the lockdown imposed by COVID‐19. Items examples are: “We communicate better” and “We spend more fun time together.” The items were used in two different ways: For descriptive purposes, we considered each item separately, whereas for inferential purposes, we used a composite index computed by avering the scores of the six items. The scale showed good internal consistency (α = .84). Higher scores indicated higher perceived positive changes in family life.

#### Parent–child relationship satisfaction (P‐CRS)

This was a one‐item ad hoc scale: “Overall, how do you consider the relationship with your children during this period?” Participants were asked to place their response on a continuum from 1 (*very negative*) to 10 (*very positive*) with 5 (*neither positive nor negative*) as a midpoint.

#### Knowing persons diagnosed with COVID‐19

This was a one‐item ad hoc scale: “Do you know someone who got sick because of COVID‐19?” (0 = *no*, 1 = *yes*).

### Data analysis

With regard to our first objective, descriptive statistics (in terms of means and standard deviations) and Spearman and Pearson correlations among the variables of interest were performed. In addition, we performed paired‐sample *t* tests to detect any differences between mean scores of perceived positive and negative changes in family life. Finally, with the aim of describing the specific positive and negative changes in family life domains, we reported the percentage of parents who scored equal to or higher than 4 on each item.

With regard to our second objective, to consider the role of the stage of family life cycle and family size for the level of perceived changes in family life, we performed a two‐way multivariate analysis of variance (two‐way MANOVA) with the stage of the family life cycle based on children's ages (stage 1 = below 13 years of age, stage 2 = between 13 and 17 years of age, stage 3 = between 18 and 34 years of age) and family size (1 = only‐child family, 2 = two‐child family, and 3 = three or more children in the family) as the between‐subjects factors. In addition, to control for P‐CRS, parents' gender (0 = men, 1 = women), and knowing persons diagnosed with COVID‐19 (0 = not knowing persons diagnosed with COVID‐19 and 1 = knowing persons diagnosed with COVID‐19), we considered these variables as covariates in the MANOVA. P‐CRS was first recoded into the two categories above or below the median (*Mdn* = 8; 0 = low levels of P‐CRS and 1 = high levels of P‐CRS). Given the large sample size, we considered as significant only those effects with *p* < .01. The Sidak post hoc test was used to explore significant differences in means. Statistical analyses were performed using SPSS 21 (IBM Corporation, Armonk, NY).

## results

In Table 1, we report means and standard deviations and Spearman and Pearson correlations.

**Table 1:** Means, SDs, and Spearman and Pearson Correlation Among the Study Variables

	1.	2.	3.	4.	5.	6.	7.	Mean (*SD)*	Range
1. Family life cycle	—							—	—
2. Family size	.18**	—						—	—
3. P‐CRS	−.05	−.02	—					—	—
4. Parent's gender	.01	−.00	.03	—				—	—
5. COVID‐19	−.02	.02	.01	−.05*	—			—	—
6. Negative changes	−.05*	.03	−.30**	.01	.00	—		2.84 (.89)	1–5
7. Positive changes	−.02	.09**	.30**	.04	−.03	−.30**	—	3.66 (.71)	1–5

*Note*. Spearman correlations are reported in italics. P‐CRS, Parent–child relationship satisfaction; COVID‐19, knowing someone diagnosed with COVID‐19.

* *p* < .05.

** *p* < .01.

**Figure 1:**
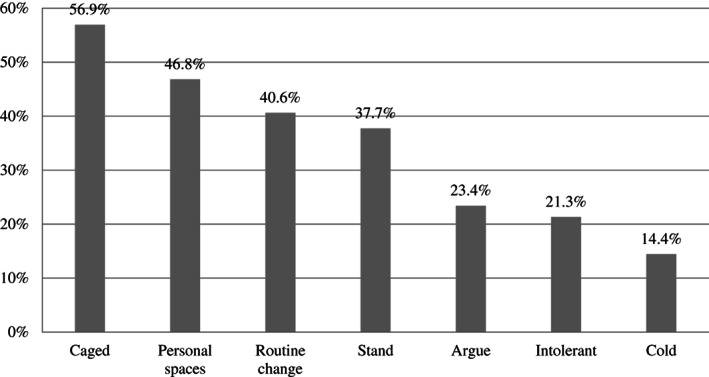
Percentages of Parents Perceiving Negative Family Changes During COVID‐19 Lockdown. *Note*. Caged = We feel caged; Personal spaces = We miss personal spaces; Routine change = We suffer because of changes to our daily routine; Stand = We can stand one another less; Argue = We argue more; Intolerant = We are more intolerant with one another; Cold = We are more “cold” and distant.

**Figure 2:**
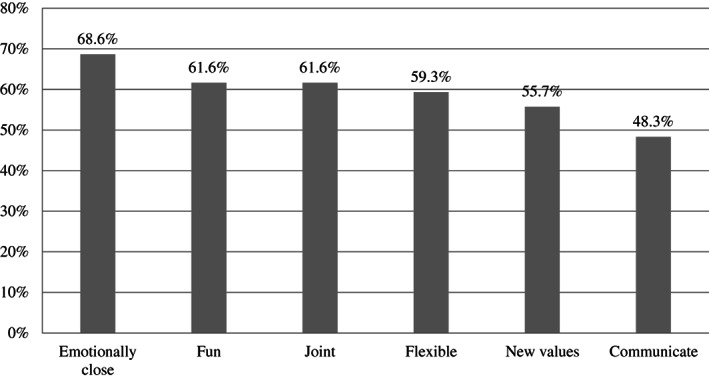
Percentages of Parents Perceiving Positive Family Changes During COVID‐19 Lockdown. *Note*. Emotionally close = We feel more emotionally close; Fun = We spend more fun time together; Joint = We engage in joint activities more; Flexible = We are more flexible in managing family life; New values = We have discovered new values; Communicate = We communicate better.

On the basis of the possible range of responses, respondents appeared to perceive a moderate to high perception of positive changes in family life and a moderate degree of negative changes. P‐CRS was moderately negatively and positively related to negative and positive changes, respectively. Negative and positive changes were negatively and moderately associated with each other. Results of the paired sample *t* test showed that in general, parents reported more positive (*M* = 3.66, *SD* = .71) than negative (*M* = 2.84, *SD* = .89) changes, *t*(1165) = −21.269, *p* < .001. Figures 1 and 2 display the percentage of parents who reported perceiving negative and positive changes in family life (score 4 = *partially agree* and 5 = *strongly agree* of the response scale) during the COVID‐19 lockdown, respectively.

Regarding MANOVA analysis, Box's test of equality of covariance was not significant. The multivariate result was significant for family size, Pillai's trace = .015, *F*(4,2,790) = 5.402, *p* = .0002, *ηp* ^2^ = .008. Neither a significant effect of the stage of the family life cycle, Pillai's trace = .009, *F*(4,2,790) = 3.020, *p* = .0169, *ηp* ^2^ = .004, nor a significant interaction effect between family size and the stage of the family life cycle, Pillai's trace = .004, *F*(8,2,790) = 0.677, *p* = .7124, *ηp* ^2^ = .002, was found.

With regard to family size, univariate testing found the effect to be significant for perceived family positive changes, *F*(2,1,395) = 6.249, *p* = .002, *ηp* ^2^ = .009, regardless of the level of P‐CRS, parents' gender, and knowing persons diagnosed with COVID‐19. Sidak post hoc tests highlighted that parents with two children (*M* = 3.70, *SD* = .03) and parents of three or more children (*M* = 3.79, *SD* = .06) reported higher positive changes compared to parents of one child (*M* = 3.58, *SD* = .03). The effect of family size turned out to be not significant for perceived family negative changes, *F*(2,1,395) = 2.741, *p* = .0650, *ηp* ^2^ = .004.

## discussion and conclusions

This exploratory study is, to our knowledge, one of the first to explore how the COVID‐19 pandemic affected family life. In particular, we investigated parents' perceptions about negative and positive changes in family life during the lockdown phase. In this study, we took into account the role of the stage of the family life cycle and family size.

On four of seven items, approximately 40% or more reported perceiving some negative changes in family life during the lockdown. The most problematic issues experienced by parents were a feeling of being caged (56.9%) and a lack of personal space (46.8%). Moreover, due to social isolation and home confinement, many parents also reported relevant changes in family daily routines and rituals (40.6%) (Figure 1). As has been reported elsewhere, people's quality of life has been profoundly influenced by the sudden spread of the COVID‐19 virus and the consequent move to lockdown (e.g., Brooks et al., 2020; Casagrande et al., 2020). More specifically, literature has shown that confinement, lack of freedom, and loss of usual routines are consequences perceived as highly distressing in similar pandemic situations (e.g., Braunack‐Mayer et al., 2009; Hawryluck et al., 2004; Robertson et al., 2004).

Interestingly, parents reported perceiving more positive than negative family changes. In line with the literature (e.g., Lades et al., 2020), our results seem to suggest that restrictions related to COVID‐19 pandemic, which determined a higher portion of time spent with children, might have produced some benefits for family well‐being. In particular, a large percentage of parents in our sample reported feeling more emotionally close to their children (68.6%) and spending more fun time with them (61.6%; Figure 2).

Research on resilience has already provided empirical evidence on the extraordinary human capacity to overcome trauma and to emerge even stronger than before (e.g., Polizzi et al., 2020). Family resilience, that is, the ability of the family, as a functional system, to withstand and rebound from adversity and to strengthen ties between family members and increase their competence to face future challenges (Walsh, 2003, 2008), appeared to play a key role in facing highly stressful situations. For instance, a study carried out with 452 American respondents after the disaster of Hurricane Katrina showed the positive relationship between family hardiness and the ability of the family to cope with the disaster (Hackbarth et al., 2012).

In this perspective, according to Prime et al. (2020), some families may experience *posttraumatic growth*, defined as the positive psychological change arising from the challenges of highly stressful situations. As stated by Tedeschi and Calhoun (2004), “post‐traumatic growth is not simply a return to baseline, it is an experience of improvement” (p. 4) and positive changes. Resilience, in fact, enables families to respond effectively to crisis situations and, as a result, to recover and grow from the experiences undergone. In the early period of the pandemic, parents responding to this research appeared to be able to find new meanings in facing the COVID‐19 pandemic, while focusing on family relationships. Therefore, dependent on family circumstances, perhaps, spending more fun time with children and engaging in more family joint activities could be considered effective coping strategies to overcome stressful situations and favor psychological well‐being, similarly to those “coping activities” (e.g., hobbies activities) already emerged as relevant in research on mass traumas (e.g., Bonanno et al., 2010).

Moreover, in line with the study by Taylor et al. (2008), our data suggest that family size could be considered a resource for family well‐being during the pandemic. Although in the literature the presence of more children in the family has been found to be related to more parenting stress because of the increased demands on resources (e.g., Gameiro et al., 2009; Hong & Liu, 2019; Östberg & Hagekull, 2000), findings from our study suggested a different trend. Parents with more than one child did not report higher negative changes in family life during the lockdown, suggesting that a higher number of children in the family does not systematically correspond to a decrease in family well‐being. Moreover, parents of more than one child reported perceiving greater positive changes in family life, regardless of the level of P‐CRS, parent's gender, and knowing persons diagnosed with COVID‐19. Parents of more than one child showed a greater relational regenerative capacity in the face of COVID‐19 lockdown and were more likely to be able to empower internal relational resources while experiencing the lockdown period. We can speculate that families larger in size are little social communities, and this may buffer the negative effect of social isolation due to COVID‐19 restrictions. Indeed, isolation is a signature of the COVID‐19 pandemic that may have detrimental effect on resilient capacities for one‐child families due to a reduced circle of social contacts.

### Limitations

This study has some limitations. First, the study had only an exploratory intent and used a cross‐sectional design. To address questions about specific family changes involved in the COVID‐19 pandemic and to provide more accurate assessment of long‐term psychological adjustment to this health crisis, longitudinal research is needeed. Furthermore, the use of self‐report measures may have led to biased reporting, and due to the novelty of the phenomena explored, we could not rely on validated measures to assess the study variables and only ad hoc items were used. It should also be noted that the P‐CRS variable referred to a global index of parent–child relationships satisfaction across families; this prohibits identification of specific child parents referred to when having more than one. Moreover, because data were collected through an online questionnaire, those who do not use network devices were excluded. Finally, results highlighted that only a small percentage of the variance was explained by the independent variables (e.g., Eta^2^ = .01).

### Implications

Despite these limitations, the main findings of this study offer some practical implications for family‐based interventions aimed at reducing the negative consequences of pandemic and at promoting resilience among parents. Research in this area can inform professionals about how to provide support and assistance to those parents coping with major life disruptions such as the COVID‐19 pandemic. In particular, our findings suggest family interventions focused on strategies aimed at contrasting negative feelings related to being caged, lacking of personal spaces, and changing family daily routines and rituals, that were perceived by the parents as the most difficult experiences during the lockdown period. Moreover, spending time with one's children and investing in family relationships, especially for families larger in size, could be considered as an effective coping strategy to deal with traumatic situations and promote positive family changes in stressful situations.

Future studies should include in the analyses other predictors of perceived family changes, as well as other potential intervening variables (e.g., parents' working conditions and parents' financial stability). Moreover, future research should be aimed at evaluating whether perceived family changes can in turn have an effect on family well‐being, relying on longitudinal data and validated measures that are more adequate to capture the complexity of family processes.
